# Adolescent HIV/AIDS: Issues and challenges

**DOI:** 10.4103/2589-0557.68993

**Published:** 2010

**Authors:** Smriti Naswa, Y. S. Marfatia

**Affiliations:** Department of Skin VD, Government Medical College & SSG Hospital, Vadodara, India

**Keywords:** Adolescent, human immunodeficiency syndrome / acquired immunodeficiency syndrome, issues

## Abstract

Adolescence (10-19 years) is a phase of physical growth and development accompanied by sexual maturation, often leading to intimate relationships. Adolescent HIV/AIDS is a separate epidemic and needs to be handled and managed separately from adult HIV. The adolescents can be subdivided into student, slum and street youth; street adolescents being most vulnerable to HIV/AIDS. Among various risk factors and situations for adolescents contracting HIV virus are adolescent sex workers, child trafficking, child labor, migrant population, childhood sexual abuse, coercive sex with an older person and biologic (immature reproductive tract) as well as psychological vulnerability. The most common mode of transmission is heterosexual, yet increasing number of perinatally infected children are entering adolescence. This is due to “bimodal progression” (rapid and slow progressors) among the vertically infected children. Clinically, the HIV infected adolescents present as physically stunted individuals, with delayed puberty and adrenarche. Mental illness and substance abuse are important co-morbidities. The disclosure and declaration of HIV status to self and family is challenging and guilt in sexually infected adolescents and tendency to blame parents if vertically affected need special consideration and proper counseling. Serodiscordance of the twins and difference in disease progression of seroconcordant twins are added causes of emotional trauma. Treatment related issues revolve around the when and what of initiation of ART; the choice of antiretrovirals and their dosages; issues related to long term ADRs; sense of disinhibition following ART commencement; adherence and resistance.

## ADOLESCENT HIV IS A SEPARATE EPIDEMIC

Adolescent HIV/AIDS is a separate epidemic and needs to be handled and managed separately from adult HIV as not only they face problems in accepting their HIV status, need for life long treatment and other positive family members but also have sad memories of their lost parent and a big question mark in the future regarding health, education, carrier and marriage. An adolescent is an individual who gets infected with HIV once but stays infected and affected for life.

## INTRODUCTION

Adolescents are defined as individuals in the 10–19 year age group. The Government of India, however, in the National Youth Policy, defines adolescents age group as 13–19 years. This phase is characterized by acceleration of physical growth and, psychological and behavioral changes, thus bringing about transformation from childhood to adulthood. Physical growth and development are accompanied by sexual maturation, often leading to intimate relationships. In addition, the adolescent experiences changes in social expectations and perceptions. The individual’s capacity for abstract and critical thought also develops, along with a sense of self-awareness when social expectations require emotional maturity.[1]

## DEFINITION AND SUBDIVISIONS

In terms of psychological, physiologic and social development, adolescence is subdivided into early, middle and late adolescence.[1] In the early stage (10–13 years), independence-dependence struggles are heralded by rapid physical changes with the onset of puberty (8–11 years in females and 9–11.5 years in males). The middle stage (14–16 years) is characterized by an increased scope of feelings, and increased importance of peer group values and more risk-taking behaviors. The late stage (17–19 years) represents emerging adults who have successfully transitioned into accepting responsibility for their behaviors.[2]

## WHY FOCUS ON ADOLESCENT HEALTH?

Adolescents constitute a considerable proportion of India’s population (22%). They are a rich human resource and an important part of the development process. Good health of adolescents will help in raising the health status of the community. Adolescents are highly vulnerable to human immunodeficiency virus (HIV)/acquired immunodeficiency syndrome (AIDS) and other sexually transmitted infections (STIs). Health of adolescent girls has an intergenerational effect.[1]

## EPIDEMIOLOGY

### Global scenario [Figure 1]

The World Health Organization estimates that 10.3 million youth aged 15–24 years are living with HIV/AIDS (most without knowing that they are infected) and half of all new infections are occurring among young people on a global basis.[5] Each year, about 4 million people younger than 20 years are diagnosed with STIs including herpes, human papillomavirus (HPV), chlamydia, gonorrhea, and the HIV.[6]

**Figure 1:**
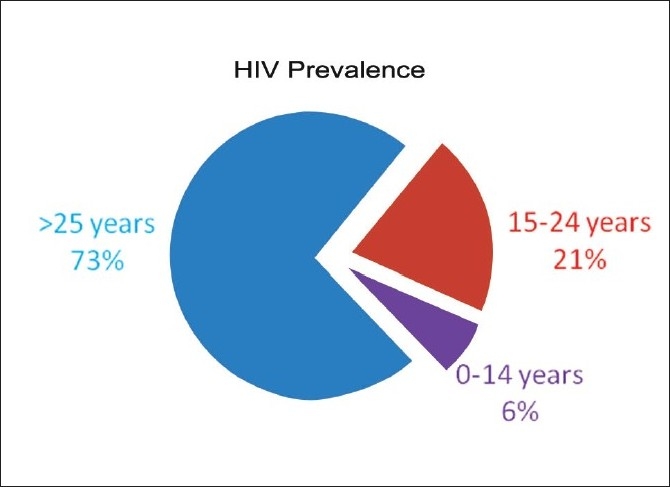
Agewise global HIV prevalence[3,4]

### Indian scenario

Adolescents constitute 22% of India’s population.[1] Fifty percent girls are married by 18 years National Family Health Survey (NFHS 2). Unmet need for contraception (15–19 years) is 27% (NFHS 2). Adolescents constitute 22% of India’s population.[1] Fifty percent girls are married by 18 years (NFHS 2). Contraceptive use in 15–24 years age group increases between NFHS-2 and NFHS-3 by more than 1% point per annum.[7] Premarital sexual relations are increasing. Trafficking and prostitution has increased. Forty percent of the adolescents start taking drugs and fall a victim of substance abuse between 15 and 20 years (UNODC, 2002).[8]

Over 35% of all reported AIDS cases in India occur among young people in the age group of 15–24 years.[1] UNICEF estimates about 4 million affected children in India, located mostly in the high HIV-burden states of south and northeast India (affected children include those living with HIV or those who are orphaned by AIDS, and children whose parents are living with HIV).[9] Majority of the adolescents are infected through unprotected sex.[1,8] A survey among injecting drug users has shown that the use of sterile injecting equipment was lower among the younger respondents (less than 19 years) compared to older respondents.[1]

Eleven percent of women and 8% of men (15–24 years) who have ever had sexual intercourse reported an STI or STI symptom in the 12 months preceding the NFHS-3 survey. Among men, self-reported prevalence of the two STI symptoms—abnormal bad smelling genital discharge and genital sore or ulcer–is higher among adolescents than among men in the age group 20–24.[7]

## KNOWLEDGE, ATTITUDE AND PRACTICES -GAP (KAP-GAP) IN ADOLESCENTS LIVING WITH HIV/AIDS

Misconceptions about HIV/AIDS are widespread.[8] UNICEF statistics (2003–2008) show that only 36% of adolescent males in India have comprehensive knowledge of HIV, while their female counterparts lag behind with just 20% of them having complete and accurate HIV information.[10] A national study by NACO/UNICEF (National Behavioral Surveillance Survey, 2001) among young people (15–24 years) found that the level of awareness about HIV was higher in urban adolescents compared to their rural counterparts, more in males than females and that education increased the levels of awareness. Eighty-three percent respondents knew of at least two correct modes of transmission of HIV/AIDS.[1] Nearly half of them report using condom in the last casual sex and consistent condom use is much lower.[1] In the UNICEF study (2003–2008), 37% males used condom at last higher-risk sex, while only 22% females had used condoms.[10]

In the US, 40 HIV+ adolescents/young adults were interviewed. HIV transmission/safer sex knowledge was low (18% at mean age 16.6 years and 28% at 18.3 years), increased with age, and both self-efficacy for and actual condom use was relatively high. Like their uninfected peers, HIV-infected youth perceive that most people of their age are having sex and that there is indeed pressure to do so, both by a certain age and at a certain point in a relationship.[11] The rates of sexual activity increased over time as the cohort aged, while condom use generally remained high (88%). Although the observation that almost one fifth of the sexually active sample had either become pregnant or gotten someone pregnant in their lifetime suggests inconsistent condom use.[12]

## PATHOGENESIS

**Figure 2:**
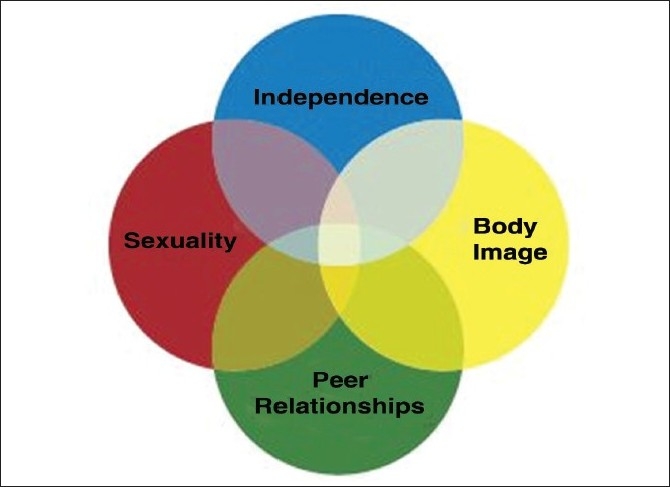
Adolescent vulnerability[2]

An understanding of the developmental pattern, though it may not be exactly the same in every adolescent, is of utmost importance for every healthcare provider to evaluate an adolescent’s behavior as it applies to their health.[2] The pathogenesis of adolescent HIV/AIDS encompasses various factors over and above the disease and its causative organism per se, which includes the physical, biological and psychological vulnerabilities as well as the special situations which various groups of adolescents have to face. [Figure 2, Table 1]

**Table 1:** Adolescent vulnerability

Behavioral vulnerability
The age of experimentation
Gender power imbalance
Adolescents going away from home for studying
High risk behavior
Biological vulnerability
Anatomical and physiological vulnerability
Economic vulnerability
Commercial sex workers
Migrant population
Child labor
Human trafficking
Social vulnerability
Early age of marriage
Early child bearing
Marriage with an older, sexually active male
Non-consensual sex
Coercive sex
Child abuse
Inadequate sex education
Limited access to information and counseling
Limited access to STD treatment facilities

## ADOLESCENTS IN SPECIAL SCENARIOS

Given the prevailing social, economic and cultural inequities in India, a large number of adolescents are forced to work and live in inhospitable, unsafe and exploitative conditions. They can be found trapped in various situations discussed below, emphasizing the need to individualize the therapy according to the background from which an adolescent comes [Table 2].

**Table 2:** Types of adolescents/youth

Category	Information about modes of transmission		Risk of HIV	Sexual activity
	Heterosexual	Homosexual		
Student youth	Knowledge present, but scanty	Knowledge present, but scanty	Minimal	Not sexually active
Slum youth	Complete information	Completely ignorant	High	Sexually very active (mostly homosexual activity)
Street youth*	Complete information	Complete information	Very high	Very active sexually

* Street youth is a term used to refer to children who live on the streets of a city. They are deprived of family care and protection. Most children are from about 5 to 17 years of age. Human Rights Watch estimates that approximately 18 million children live or work on the streets of India.[13] Street youth has complete information on all modes of transmission and their risk of acquiring HIV is very high compared to other two groups because of the environment they live in. Crime, prostitution, gang-related violence and drug trafficking,[13] sexual exploitation, unprotected sex with multiple sex partners, sex at young age, and coercive sex by older peers or adults are normal in their lives[14]

### Orphaned children

Children orphaned by AIDS are those under the age of 18 who have lost one or both parents to the disease. According to UNAIDS, nearly 15 million orphan children are there worldwide.[15] It is estimated that India has the largest number of AIDS orphans of any country and this number is expected to double in the next 5 years.[13] World Bank estimates suggest that the number of children in India orphaned by AIDS was 2 million in 2005, which is expected to double by 2010 and remain exceptionally high until 2020 or 2030.[16,17]

The odds against AIDS-orphaned children are staggering. Stigmatized and discriminated, though no fault of theirs, they are psychologically distressed, and they do not have access to basic education and basic health care. These AIDS orphaned children are far more vulnerable to abuse and all forms of exploitation like prostitution, beggary, juvenile delinquency, and drug abuse.[18] Another worrying phenomenon is the emergence of child-headed households. Children whose parents have AIDS and/or die with AIDS, are thus impacted medically, socially, and economically.[17]

### Commercial sex workers

Conservative estimates state that around 300,000 children in India are engaged in commercial sex.[13] Of the 1588 female sex workers (FSWs) interviewed, “Devadasi” FSWs (socially accepted FSWs) (26% of total) had initiated sex work at a much younger age (mean 15.7 vs. 21.8 years).[19] A random sample of 28 out of 86 brothels along the G. B. Road in India revealed that almost 60% of the prostitutes were children.[20,21] Indian legislation offers protection to children in difficult circumstances but it is often hard to ensure its enforcement. UNICEF itself admits “for adolescents who have been commercially sexually exploited or whose parents are engaged in commercial sex work, options for protection and development are scarce.”[22]

### Child trafficking

It is estimated that 200,000 persons are trafficked in India every year. Only 10% of human trafficking in India is international, while almost 90% is interstate. Nearly 40,000 children are abducted every year, of whom 11,000 remain untraced according to a report by the National Human Rights Commission of India.[23] Adolescents, especially girls, mostly from disadvantaged communities and families, are trafficked for the purposes of early, forced marriages, for domestic labor (unpaid or poorly paid), for commercial sex work and are forced to work in inhospitable, unsafe and exploitative conditions.[22]

### Migrant population

"“Being mobile in and of itself is not a risk factor for HIV infection. It is the situations encountered and the behaviors possibly engaged in during mobility or migration that increase vulnerability and risk regarding HIV/AIDS.”[24]

Some of the migrant adolescents are members of families and communities living in remote areas with few, if any, livelihood options that are socially and economically marginalized. Others are part of units that are on the move—caught up in unrelenting cycles of migration in search of work. Roughly, 27% of the country’s population is considered migrant and up to 77% of this proportion is women and children. These adolescents tend to suffer from lack of education and often lose almost all access to basic services.[22] They have little access to HIV/STD information, voluntary counseling and testing and health services.[24,25]

### Childhood sexual abuse

Childhood sexual abuse (CSA) leads to increased adolescent sexual vulnerability. In a study in New Zealand on 520 young women (14–18 years), those reporting CSA, and particularly severe CSA involving intercourse, had significantly higher rates of early onset consensual sexual activity, teenage pregnancy, multiple sexual partners, unprotected intercourse, sexually transmitted disease, and sexual assault after the age of 16. The reasons are firstly, exposure to CSA was associated with a series of childhood and family factors including social disadvantage, family instability, impaired parent child relationships, and parental adjustment difficulties that were also associated with increased sexual vulnerability in adolescence. Secondly, there appeared to be a causal chain relationship between CSA and sexual experiences in which CSA was associated with early onset sexual activity which, in turn, led to heightened risks of other adverse outcomes in adolescence.[26]

### Coercive sex

Coercive experiences at sexual debut have been shown to be associated with other sexual risks throughout the life course. In interviews conducted with girls from 12 to 19 years in sub-Saharan Africa in 2004, it was revealed that there are four primary types of sexual coercion: forced sex; pressure through money or gifts; flattery, pestering, and threatening to have sex with other girls; and passive acceptance. Reproductive health risks correlated with sexual coercion include STIs (which can cause cervical cancer and infertility) including HIV, unintended pregnancy which can possibly lead to unsafe abortion, and the onset of risk-taking behaviors including other nonconsensual sexual experiences, multiple partnerships, and unprotected sex. A negative sexual experience can also result in a host of negative psychological outcomes including sexual dysphoria, anxiety, eating disorders, substance abuse, depression and even suicide or attempted suicide.[27,28]

## BIOLOGICAL VULNERABILITY OF ADOLESCENTS

### Anatomic and physiologic changes

The cervix in adolescent females has areas of exposed columnar epithelium which gets fully covered with squamous epithelium only in adulthood. The presence of exposed columnar epithelium is called as ectopy, making the adolescent increasingly vulnerable to STDs. *Chlamydia trachomatis*, and *Neisseria gonorrhoeae*, for instance, infects columnar, not squamous epithelium.[29] Ectopy may also contribute to HIV acquisition and HIV shedding.[30] The risk is particularly high when sexual debut occurs early and may be amplified when sex is not consensual or when it is with older partners. Many of these factors put young women at higher risk, as reflected in the earlier rise in HIV infection rates in young women than in young men.[31] Lower prevalence of H_2_O_2_ producing organisms in early adolescence, leads to higher vaginal pH making the adolescent more susceptible to STDs. 30 Also, mucus is thinner in early adolescence than adults or older adolescents, which permits organisms to penetrate more easily and to attach to mucosal sites.[30]

## PSYCHOLOGICAL AND COGNITIVE VULNERABILITY

The early adolescents (10–13 years) use a concrete style of reasoning, focusing on the present only, not thinking into future consequences, and hence their actions do not take into account STDs like chlamydia and HIV which do not manifest in them in a decade or so.[30]

Secondly, adolescence is a growing and learning phase; hence, it is difficult for them to correctly implement complicated tasks such as condom use.[30]

Third problem is the peer pressure acting synergistically with the “risk seeking behavior” leading to unsafe sex, casual sex, experimentation with sexuality, drug abuse including intravenous drugs, all directing toward increased exposure to risk of HIV transmission.[30]

Another issue is the response and attitude of family, teachers and society toward adolescents, who would not give them the basic information about sex, sexuality and HIV/AIDS and other health-related issues.[30]

In a study conducted on 40 adolescents, HIV positive youth appeared to have a later onset of sexual risk behavior than a normative sample of U.S. high school students. This may be a result of delayed emotional maturity resulting from reduced expectations of survival and independent functioning and therefore greater dependence on their families (Battles & Wiener, 2002)^12^, yet, it is not known whether these pre-pubertal youth engage in sexual risk behaviors.

This suggests that interventions designed to reduce the risk of sexually transmitting HIV by this population require developmentally appropriate psychological and social approaches that target perceptions of peer influence and emotional well being.[12,32]

## HIV IN ADOLESCENTS

### Modes of transmission

Exposure of HIV-infected adolescents and young adults to the virus through sexual intercourse is the most common mode of transmission. In addition, there are an increasing number of children who were infected as infants who are now surviving to adolescence. Toll of adolescents being exposed to this virus by injection drug use is also on the rise.

In a study conducted in the Department of Skin VD, Government Medical College, Vadodara, 31 HIV+ adolescents (20 males and 11 females) were enrolled over 2 years. Vertical transmission was the commonest mode (64.5% cases), suggesting at least one or two more cases in the family. Four cases (13%) acquired infection via sexual route, emphasizing the importance of adolescent sex education.[33]

### Adolescent HIV: Clinical manifestations

The perinatally infected children, as the immune system weakens, grow slowly and become vulnerable to recurrent infections and illnesses.[6] Hence, as they become adolescents, they are already physically stunted and vulnerable to innumerable infections. Many HIV-infected children, especially those with low CD4 counts, do not mount protective antibody response against measles even after proper immunization, and thus continue to be susceptible in their adolescence and later adulthood.[34]

The immune system of the adolescents provides a more robust response to HIV, especially when started on antiviral medications, as compared with adults. Data from Pediatric AIDS Clinical Trials 381 (PACTG 381) demonstrated adolescents to have relatively better immune reconstitution after 3 years of highly active antiretroviral therapy (HAART) in comparison to that of adults. Also, there was a trend toward higher virus loads in males, compared with females at comparable CD4 counts. In addition, higher rates of persistence of HPV are noted in adolescent women with HIV.[29]

In a 5 year study of 983 HIV-infected children aged 6 to 18 years in US, it was found that HIV-infected children may experience delayed puberty and adrenarche compared with similarly aged HIV negative children. Thus, immunosuppression was associated with delayed pubertal onset in perinatally HIV-infected children.[35]

Poor growth is reported in as many as 50% of HIV-infected children. HIV infection leads to significantly lower mean birth weight and length. Pediatric HIV further reduces birth weight. Also, there is deficiency of several micronutrients, especially vitamin A. Progressive stunting, that is, proportionately decreased linear and ponderal growth, appears to be the most common abnormality in perinatally infected children and adolescents and is accompanied by preferential decreases of fat-free or lean body mass.[36] This stunting and growth retardation has a huge psychological impact on adolescents for whom the “body image” is one of the most important issues in life.

In the study of Department of Skin VD, Vadodara, 61% cases presented when they had developed AIDS, while only 13% appeared at an asymptomatic stage, which warrants the urgent need to early diagnosis in all suspected cases, which provides care givers an opportunity for a timely intervention to prevent developmental delay and other HIV-related complications.[33] More and more ways to diagnose the disease early should be sought to prevent developmental delay; and organ-specific manifestations like cardiomyopathy, nephropathy etc which are a direct consequence of HIV-1/ autoimmunity.[37] In addition to this, early HAART may have a therapeutic effect in improving or preventing these manifestations.[38]

The opportunistic infections (OIs) like toxoplasmosis, cryptococcosis and disseminated fungal disease which are commonly seen in adults are relatively scarce in children below 8 years of age because of the lack of exposure of children to the etiologic agent, but they become common in adolescence.[34]

Mental illness and substance abuse are important co-morbidities for HIV+ adolescents, and failure to identify and address these issues will prevent adolescents from successfully coping with their illness or adhering to antiretroviral treatment. In the Reaching for Excellence in Adolescent Care and Health (REACH) study, 14% of females and over 25% of males reported drinking alcohol during the past 3 months, and 7% of females and 20% of males reported using hard drugs during the same period. Substance abuse treatment and mental health care are integral components of comprehensive care.[39]

## DISEASE PATTERN AND PROGRESSION

In 1996, REACH recruited 300 HIV-positive and 150 HIV-negative adolescents aged 12–19 years to examine unique features of adolescent HIV disease progression and manifestations; the majority of youth had acquired their infection sexually during adolescence and enter care asymptomatic but with moderate immune dysfunction (median CD4 count: 410 cells/mm^3^). In contrast, perinatally infected children who survive into adolescence usually have advanced disease, with 63% having AIDS, but 20% having CD4 > 500/mm^3^. It also reported a newly emerging group of congenitally infected adolescents who are first being diagnosed in adolescence. This highlights the importance of offering HIV testing to all children of HIV+ parents.[39]

There is a “bimodal progression” (rapid and slow progressors) in vertical transmission cases. About 20% of children have early onset of symptoms before the advent of effective therapy. These children have a rapid downhill course in the first 12 months of life, marked by rapid decline in CD4 counts, development of category C disease, or death. Thus, many of them do not enter adolescence. There appears to be a number of predictors of rapid progression, including severe maternal disease, evidence of *in-utero* transmission, early hepatosplenomegaly, and higher viral loads after 1 month of life.[40] Slow progression in spite of high prevalence of anemia, malnutrition and infectious diseases (TB) in our country[33] on the other hand, can have various factors like genetic, viral strains, implementation of prevention of parent to child transmission (PPTCT), mode of delivery, breast feeding and whether antiretroviral therapy (ART) was taken or not, playing a role.

In the study conducted in Medical College, Vadodara, although a very small study to make generalization, it was found that mean time for disease progression in vertically transmitted cases was 12.5 years, who thus being “slow progressors” presented in adolescence. Blood transfusion cases presented with clinical illnesses in 7 years duration, while adolescents who contracted HIV through sexual route were the most rapid progressors of mean duration only 2.5 years. The concomitant presence of STIs could have been one of the causes of such fast progression.[33]

## DISCLOSURE AND DECLARATION OF HIV/AIDS: CONSENT AND CONFIDENTIALITY ISSUES

Disclosure of HIV/AIDS to self and to parents has multifaceted challenges. The adolescent is an emotionally vulnerable age group, and the way in which they will respond to their disease status can never be predicted. On one hand, where sexually infected ones can find it difficult to face their family due to guilt, the perinatally affected adolescents, on the other hand, can be expected to blame their parents for their situation.

Many young people do not access needed healthcare services for fear of disclosure to parents/guardians. When providing care to minors, there should be a discussion of how confidentiality issues are handled. Hence, the need arises for intelligently and patiently tackling the situation, with the active participation of health care givers, family as well as society.[29]

## ISSUES RELATED TO TWINS

In the study in Vadodara, in the seroconcordant twins, the first born had developmental delay as well as faster HIV progression as compared to the other twin. In the discordant twins, the first born was HIV+, whereas, the second born had escaped HIV infection. These situations of discordance of HIV status among twins worsen the emotional trauma.[33]

## TREATMENT

Is long-term ART ultimate?

- The ART leads to increased immunity and thus increased survival from childhood AIDS, thus more and more positive children enter adolescence.
- *Disinhibition:* ART initiation will give false sense of security to adolescents leading to high-risk sexual behavior.
- Differences in drug dosing-issues
  1. Adult guidelines for antiretroviral therapy are usually appropriate for postpubertal adolescents, because HIV-infected adolescents who were infected sexually or through injection drug use during adolescence follow a clinical course that is more similar to that of adults.[41]
  2. Because puberty may be delayed in perinatally HIV-infected children, continued use of pediatric doses in puberty-delayed adolescents can result in medication doses that are higher than the usual adult doses.[41]
  3. The pharmacokinetics of some medications change during adolescence (especially for hepatic enzyme inducers/inhibitors and protein-bound medications). No clinical impact has been noted for the nucleoside analogues, to date. Less information is available for nonnucleoside reverse transcriptase inhibitors and protease inhibitors.[39]
  4. Because of the concern that pubertal changes may affect pharmacokinetics, dosing is based on the Tanner puberty stage and not age. Pediatric dosing should be used for adolescents who have entered puberty or are early in puberty (Tanner stage I/II). Dosing for adolescents who are in the middle of puberty (Tanner stage III/IV) should be based on whether they have completed their growth spurt. Adolescents who have completed puberty (Tanner stage V) should be given adult doses.[39,42]
  5. Life-long treatment with monitoring- Issues such as toxicity, pill or liquid volume burden, adherence, and virologic and immunologic parameters should be considered in determining when to transition from pediatric to adult doses. Therapeutic drug monitoring can be considered in selected circumstances to help guide therapy decisions under this context.[41]
- Long-term adverse drug reactions (ADRs): ART-related long-term metabolic and morphologic ADRs have tremendous psychological impact and effect on “body image”.
- Adherence
  It is well recognized that maximal adherence to the prescribed antiretroviral (ARV) regimen is crucial for achieving and maintaining optimal antiretroviral response. In a REACH project of 114 HIV-infected adolescents from 13 U.S. cities, only 28.3% of adolescents reported taking all of their prescribed antiretroviral medications in the previous month.[43] Adherence seems to vary with route of HIV infection. In one study, more than 90% of those infected perinatally or via blood products reported taking pneumocystis carinii pneumonia (PCP) prophylaxis, compared to only half of those with sexually acquired infection.[39]
  According to the U.S. Department of Health and Human Services (DHHS) Panel on ART Guidelines, many adolescent HIV patients experience problems adhering to HIV treatment regimens, including
  - medication related side-effects;
  - denial and fear of their HIV infection;
  - misinformation;
  - distrust of the medical establishment;
  - fear and lack of belief in the effectiveness of medications;
  - low self-esteem;
  - unstructured and chaotic lifestyles and refusal to take medication due to rebellious behavior;
  - lack of familial and social support; and
  - unavailable or inconsistent access to care or health insurance and incumbent risks of inadvertent parental disclosure of the youth’s HIV infection status if parental health insurance is used.
  Adolescents often benefit from treatment reminders (such as pill boxes, beepers, or timers).[6,41,44] A nonjudgmental, trusting relationship between the teen and the provider is crucial at this stage. In addition, the recognition and management of the HIV-associated myriad of psychosocial and mental health issues is another important facet, which has a major impact on adherence, and must be addressed for successful management of HIV.[42]
- *Resistance*: A recent study conducted by the Adolescent Medicine Trials Network for HIV/AIDS Interventions (ATN) identified primary genotypic resistance mutations to antiretroviral medications in up to 18% of the recently infected youth.[41] The resistance leads to need to switch over to second generation ART drugs, which have their own issues on efficacy and safety.
- *When to start HAART*: A REACH study suggests that patient health status, depression and perhaps living with a parent and disclosure of HIV status to parents, would be associated with HAART prescription.[39]

## TREATMENT OF OPPORTUNISTIC INFECTIONS

According to the U.S. antiretroviral guidelines, Tanner stage of the adolescent is followed to adjust the dosing of antibiotics for opportunistic infections.[6]

## IMMUNIZATION

HIV-positive adolescents need more immunizations than do HIV-positive adults. Although contacts should receive it, varicella-zoster vaccine is not currently recommended for any HIV-positive persons.[Table 3].

**Table 3:** Immunizations for adolescents[39]

Measles, mumps, and rubella (MMR) booster Diphtheria-tetanus toxoid (dT) booster Hepatitis B virus (HBV; 3 in series) Hepatitis A virus (HAV; 2 in series; not routine; recommended for men who have sex with men) Influenza (once yearly) Pneumococcal polysaccharide vaccine (1 dose) *Haemophilus influenzae* B (HIB; 1 dose; optional)^39^ Conjugated meningococcal vaccine- Menactra (optional) HPV vaccine - It can be administered to females regardless of CD4%. Recommendation: females at age 11-12 years at 0, 2 and 6 months (minimum age 9, maximum age 26 years)[45,46}

## CONCLUSION

“Enable adolescents to protect themselves and become advocates for HIV prevention.”[1]

HIV-infected adolescents face unique challenges when it comes to accepting and treating their diseases. Many adolescents are in denial, afraid, misinformed or lack familial or social support. Therefore, this age group may benefit from counseling services and supportive care. Many NGOs and help groups and help lines are working in India.

Providing care to adolescents is a multifaceted process in that no two adolescents are the same, but they all require sensitive, flexible, culturally and developmentally appropriate care. For clinicians caring for adolescents, it is crucial to understand that for the eventual success of treatment, it is critical to manage the “whole” adolescent within the context of his/her own economic, cultural, psychological, and family environment.

Reaching youngsters at an impressionable age before they become sexually active can lay the foundations for a responsible lifestyle, including sex and marriage.[47] Right information, an enabling environment and supportive services help adolescents take informed decisions regarding important health issues and contribute to a better future.

“Adolescent HIV/AIDS is an epidemic with difference and its control needs to be adolescent specific.”

## Multiple Choice Questions

Q.1. As per definition, adolescents fall in the age group of

- 10–19 years
- 13–19 years
- 15–19 years
- 11–18 years

Q.2. Adolescents constitute _____% of Indian population

- 20
- 22
- 25
- 34

Q.3. What percentage of our country’s population is migrant?

- 25
- 27
- 28
- 30

Q.4. What is cervical ectopy?

- Area of exposed stratified squamous epithelium
- Area of exposed columnar epithelium
- Area of stratified squamous epithelium covering columnar epithelium
- Area of columnar epithelium covering stratified squamous epithelium

Q.5. Adolescent vagina is more susceptible to STDs because

- Lower prevalence of H_2_O_2_ leading to lower vaginal pH
- Higher prevalence of H_2_O_2_ leading to lower vaginal pH
- Lower prevalence of H_2_O_2_ leading to higher vaginal pH
- Higher prevalence of H_2_O_2_ leading to higher vaginal pH

## Answers

Q.1. a) 10–19 years

Q.2. b) 22%

Q.3. b) 27%

Q.4. b) Area of exposed columnar epithelium

Q.5. c) Lower prevalence of H_2_O_2_ leading to higher vaginal pH
